# The level of recognition of physical symptoms in patients with a major depression episode in the outpatient psychiatric practice in Puerto Rico: An observational study

**DOI:** 10.1186/1471-244X-5-28

**Published:** 2005-06-20

**Authors:** Jorge M Tamayo, Karis Román, Juan J Fumero, María Rivas

**Affiliations:** 1 Lilly Research Laboratories, Eli Lilly and Company, San Juan, Puerto Rico; 2 Department of Psychiatry, Medical Sciences Campus, University of Puerto Rico, San Juan, Puerto Rico; 3 Department of Biostatistics & Epidemiology, Medical Sciences Campus, University of Puerto Rico, San Juan, Puerto Rico; 4 Department of Psychiatry, San Juan Bautista University, Caguas, Puerto Rico; 5 Department of Internal Medicine, Medical Sciences Campus, University of Puerto Rico, San Juan Puerto Rico

## Abstract

**Background:**

This study was designed to evaluate the psychiatrists' level of recognition of somatic symptoms associated to a major depressive episode (MDE) (DSM-IV-TR criteria) and the impact of those somatic symptoms on the treatment effectiveness.

**Methods:**

This non-interventional study was conducted in 25 medical offices in Puerto Rico from February to December 2003. It had 2 visits separated by 8 weeks. The level of recognition was determined by: the correlation between the physician clinical evaluation and their patients' self-evaluations through different validated instruments using kappa statistics. Chi-square test was used to evaluate the impact of somatic symptoms on treatment antidepressants' effectiveness.

**Results:**

All the 145 recruited patients reported the presence of at least one somatic symptom associated with their current MDE. In the two visits covered by the study, a fair agreement between the psychiatrists' and the patients' reports was noted for headache, abdominal pain and upper limb pains (0.4003 ≤ κ ≥ 0.6594). For other painful symptoms and painless somatic symptoms, the Kappa values obtained were non-significant. Slight but significant reductions in depression and painful symptoms severity were observed after 8 weeks of treatment. A proportional relationship between the pain and depression severity was observed (p < 0.0001).

**Conclusion:**

The study results show that somatic symptoms: are very common in depressed Puerto Rican patients; are significant under-reported by psychiatrists; and have a significant impact on the antidepressant effectiveness.

## Background

Although recent epidemiological studies have not demonstrated any differences in the prevalence rate of major depressive disorders in the Latino and Caucasian populations living in the United States [1], the Hispanic Health and Nutrition Examination Survey (Hispanic HANES), when compared to the Epidemiological Catchment Area (ECA) Study, showed that Puerto Ricans living in the United States have the highest prevalence of depressive symptomatology, followed by Caucasians, Mexican-Americans and Cuban-Americans [2-4]. Unfortunately, little is known about the prevalence of major depression in adults living in the island of Puerto Rico. Data suggest lower lifetime prevalence rates (4.3% – 4.6%) [5,6] in comparison to the US mainland (16.2%) [1].

Traditionally, diagnostic classification systems have focused on the emotional symptoms of a major depressive episode (MDE), such as depressed mood, markedly diminished interest or pleasure in almost all activities, and feelings of worthlessness, among others [7]. Nevertheless, the importance of somatic symptoms, also known as physical symptoms, in depressed patients has been well established. It is estimated that 69% to 92% of the patients have somatic symptoms [8-11]. Studies conducted in primary care settings [12-18] have shown significant association between major depression and painless somatic symptoms such as: vague and exaggerated multiple somatic complaints (usually more than three), fatigue, weakness, non-specific and painless musculoskeletal problems, sensations of heaviness or lightness in at least one part of the body, gastrointestinal dysfunction, shortness of breath, palpitations, dizziness, double vision, changes in sleep patterns and appetite, and polyuria. Similarly, significant association between major depression and painful somatic symptoms such as joint pains, lumbar pain and headache has also been reported [12-18]. In some cases, these somatic symptoms constitute the principal reason for consultation rather than the emotional symptoms [19]. Moreover, some studies have shown that the presence of somatic symptoms contributes greatly to the recurrence of another new depressive episode several years later [15,20-24]. In addition, this association apparently does not depend on social or economic factors such as gender, income level, education or age, but it is rather inherent to the condition just as emotional symptoms are [25-27].

Somatic symptoms have been described as part of a cultural language of affective disorders that, if misinterpreted by the clinician, can lead to unnecessary diagnostic procedures or to inadequate treatment [28]. Studies with depressed patients treated in a primary care setting show that somatic and anxiety symptoms are often overlooked in the diagnosis of depression despite such symptoms contributing significantly to detecting the disease, according to a recent logistic regression analysis [29]. In a study with primary care treated outpatients (n = 1, 456), 70% of whom were Latinos, the prevalence of somatization was 22%. Of the sub-sample with somatization, 35% had a Major Depressive Disorder (MDD) compared to only 13.7% for those without somatization (p < 0.0001) [30]. Studies comparing Latino populations with MDD residing in Colombia, Peru and Puerto Rico versus non-Latino populations with MDD in the United States, show generally higher indices of somatization despite similar rates of depressive symptoms [25,31,32].

However, very little is known about the role of somatic symptoms in populations of depressed patients treated by psychiatrists. It is possible that this group of physicians fails to recognize, value, or else underestimates the presence of these type of symptoms as an integral part of major depression and, therefore, does not monitor their evolution during the course of treatment. Some unanswered questions are: prevalence of somatic symptoms in depressed patients evaluated by psychiatrists, level of recognition of such symptoms by psychiatrists, clinical relevance of somatic symptoms in a psychiatric practice, impact of somatic symptoms on recovery and remission in depressed patients treated with different antidepressants, differences between available antidepressant medications in the improvement of painful or painless somatic symptoms.

In an effort to clarify some of these questions, we conducted an observational study in Puerto Rico. Our research method allowed for the collection of data from a representative group of psychiatrists on their level of recognition of painful or painless somatic symptoms, compared to the reports of their patients with a MDE without interfering with their usual clinical practice.

## Methods

### Selection of patients

The recruiting phase took place between February and December 2003 in 25 private outpatient psychiatry practices geographically distributed throughout Puerto Rico. Male and female subjects over 21 years of age diagnosed with MDE according to the Diagnostic and Statistical Manual of Mental Disorders, 4th Edition, Text Revision (DSM-IV-TR) were eligible for the study. Patients were excluded from the study if they had participated in clinical studies thirty (30) days prior to the first study visit. They were also excluded if they were hospitalized or met the diagnostic criteria for: refractory depression, defined as poor response to two or more appropriate antidepressant treatments for at least 12 to 16 weeks, according to the guidelines published by Souery et al. [33]; bipolar disorders; psychotic disorders; dementia; secondary depression; or painful or painless somatic symptoms of known etiology.

### Study design

Before beginning the study, the protocol was reviewed and approved by an institutional review board (IRB). Before being subjected to any study procedure, the study subjects signed an IRB approved informed consent form with a detailed explanation of the procedures and risks involved in the study.

Since this study was non-interventional, it did not contain requirements or recommendations on antidepressant or other treatment. The participating psychiatrists were free to choose the type and course of treatment for each of their patients to the best of their own clinical judgement and in accordance to their usual practice. There were no restrictions concerning the use of other therapies concomitantly with the antidepressant medication. Because the investigation sites were the psychiatrists' own private medical offices, research tools that interfered minimally with the usual clinical practice were utilized to collect the study data.

### Primary and secondary measures

The presence, severity, and number of somatic symptoms were recorded during two study visits separated by an 8 week interval, by means of the trained psychiatrists own reports and a case report form with a table to list: all the somatic symptoms asked to or reported by the patients during the last 4 weeks, and the level of severity of each one of them (1 = mild; 2 = moderate; 3 = marked; 4 = severe). The patients, on the other hand, used a self-evaluation scale: the Somatic Symptom Inventory (SSI) [34]. The SSI is a 26-item questionnaire. In this inventory, the patients' degree of discomfort for each symptom is rated from 1 to 5 (1 = absent; 3 = moderate; 5 = a great deal). All the investigators received a structured training before the first patient visit. This training included a review of the protocol, the case report forms, and the SSI scale. With respect to the SSI, the psychiatrists were instructed to use the scale as a general reference during the patient interview and somatic symptom recording but not to follow the scale's items or language literally because this could affect the patient's spontaneous response to the self-administered SSI at the end of the visit.

We categorized total SSI scores as minimal (= ≤ 52) and moderate to high (= ≥ 52) according to the patients' degree of somatic symptom discomfort. This post-hoc cut-off point was arbitrarily determined based on the high number (mean of 14) of reports by patients of somatic symptoms causing at least some degree of discomfort. Internal analysis supported this cut-off point to statistically differentiate between two groups of patients in terms of depression severity and other variables discussed below. We also conducted subscale analyses for both painless (SSI items 1, 4–8, 10–13, 15–18, 20–26) and painful (SSI items 2,3,9,14,19) somatic symptoms (Table 4). Because the SSI that we used only included 5 painful somatic symptoms, limiting the options to detect any difference between the patients' and physicians' reports, we decided to ask the patients with painful symptoms to specify the location of their symptoms with the help of human silhouettes and to rate the impact of the treatments received on the magnitude of the pain using a Visual Analogue Scale (VAS).

**Table 4:** Somatic symptoms (SSI) scores: degree of discomfort analysis (V2 vs V1) (n = 88)*

**SSI Symptom**	**SSI Score (Visit 1)**	**SSI Score (Visit 2) ‡**	**p-Value**
1. Nausea and vomiting	1.8	1.6	NS
2. Muscles soreness	2.3	2.1	<0.05
3. Pains or cramps in your abdomen	2.0	1.8	NS
4. Feeling faint or dizzy	2.4	2.0	<0.05
5. Trouble with your vision	2.2	2.1	NS
6. Muscles twitching or jumping	3.1	2.6	<0.05
7. Feeling fatigued, weak, or tired all over	3.4	2.9	<0.05
8. A fullness in your head or nose	2.6	2.3	NS
9. Pain in your lower back	3.2	2.6	<0.05
10. Constipation	2.4	2.2	NS
11. Trouble catching your breath	2.1	1.9	NS
12. Hot or cold spells	2.4	2.3	NS
13. A ringing or buzzing in your ears	2.0	1.8	NS
14. Pains in your heart or chest	2.3	1.9	<0.05
15. Difficulty keeping your balance while walking	2.2	1.9	NS
16. Indigestion, upset stomach, or acid stomach	2.7	2.4	NS
17. The feeling that you are not in as good physical health as most of your friends	3.4	2.9	<0.05
18. Numbness, tingling, or burning in parts o your body	2.7	2.2	<0.05
19. Headaches	3.3	2.6	<0.05
20. A lump in your throat	2.2	2.0	NS
21. Feeling weak in parts of your body	2.9	2.4	<0.05
22. Not feeling well most of the time in the past few years	3.4	2.9	<0.05
23. Heavy feelings in your arms or legs	2.9	2.6	NS
24. Your heart pounding, turning over or missing a beat	2.4	2.0	NS
25. Your hands and feet not feeling warm enough	2.2	1.8	NS
26. The sense that your hearing is not as good as it used to be	2.4	2.1	NS
Painless subscale†	51.6	52.5	NS
Painful subscale	13.0	11.0	<0.05
Total SSI	63.4	56.8	<0.05

*SSI – Somatic Symptom Inventory (degree of discomfort: 1 = absent; 2 = a little bit; 3 = moderate; 4 = quite a bit; 5 = a great deal); ‡ 8 weeks after Visit 1; † Somatic painless subscale correspond to the SSI items: 1, 4–8, 10–13, 15–18, 20–26.

NS = Non significant

The effects on the emotional and somatic symptoms of antidepressant therapy, as selected by the psychiatrists, were evaluated after the 8-week interval between the two study visits. The Clinical Global Impression – Severity (CGI-S) scale was used by the psychiatrists to evaluate any changes in the severity of the MDE. Also, characteristic major depressive episode emotional symptoms such as depressed mood, guilt-related thoughts, feelings of worthlessness, anhedonia, psychomotor agitation or retardation, loss of concentration, anxiety, psychotic symptoms, and suicide behavior or thoughts, were each analyzed individually according to their clinically rated severity (1 = absent; 3 = moderate; 5 = severe). Patients evaluated also the impact of antidepressant therapy in their MDE using the Patient Global Impression – Improvement (PGI-I) and a VAS for assessment of depression. Additional information on the employment status, drug abuse or dependency, concomitant therapies used to manage somatic symptoms, and the efficacy of these therapies was also collected.

### Statistical methods

The study was designed with a power of 90% to detect average differences in the level of recognition of at least one somatic (physical) symptom greater or equal to 23% by both groups analyzed, psychiatrists and patients, according to the estimate obtained using the least squares method, and based on previous data from studies on the recognition of symptoms in primary care patients.

Correlation analyses used to determine the level of agreement between the psychiatrists' evaluations and patient self-evaluations were conducted using Kappa statistics. Unanswered questions were not considered in the analysis. Symptoms were classified using a sensitivity analysis, considering the patients' report as point of comparison: >0.75, high degree of agreement beyond chance; 0.40 to 0.75, fair agreement beyond chance and <0.40, low degree of agreement.

The comparison of the number of symptoms reported by the psychiatrists versus the patients was performed using the statistical t test for independent samples. Secondary analyses were conducted to evaluate any significant statistical differences in clinical and demographic variables between genders, age groups, employment status, type of antidepressant medications, and the severity of the depression. The chi-square test for variable categories was used for this analysis.

Lastly, other secondary analyses were conducted: possible relationships between emotional and physical symptoms reported by patients and clinicians, the level of recognition of emotional symptoms by psychiatrists as compared to their patients' reports, and associations between different symptoms and response levels to the different antidepressants. The level of significance was set at P = 0.05. In order to describe the demographics of the study population, a univariate analysis was conducted by calculating the following descriptive statistics: median, variance and standard deviation.

## Results

### Patient characteristics

A total of 145 patients were evaluated in the initial visit. Table 1 presents the demographic values of the study population. Of the 145 patients that entered the study, 129 completed the procedures for visit 1 and 42 of them withdrew from the study before visit 2. The most common causes for withdrawing were: consent withdrawal (13.2%), visit 2 outside the stipulated protocol window (9.3%), researcher's decision (2.3%), and patients' change in provider (1.6%). None of the patients withdrew from the study because of adverse events or reasons related to the medications prescribed by their physicians. This report includes all the patients who completed at least the initial visit. For the analysis comparing results at visit 2 versus visit 1 we used the data of the 87 completers.

**Table 1:** Demographic variables of the study population (initial visit)

**Variable**	**N**	**Value or % (SD)**
**Average Age **(st. dev.)	145	44.5 (11.77)

**Gender: **Female	113	77.93%

**Race and Ethnicity**		
Hispanic (Latino)	145	100%
Caucasian	0	
African-Caribbean	0	
Other	0	

**Employment Status**		
Employed	49	33.8%
Housewife	6	4.1%
Student	29	20%
Unemployed	1	0.01%
Unable to work	60	41.4%

For almost half the patients in the study, the clinicians prescribed concomitant medications in addition to the antidepressants they had selected. The concomitant medications most frequently reported were anxiolytics (37.9% of the patients), antipsychotics (10.6%), tricyclic antidepressants (13.6%) and analgesics (9.1%). At baseline, alcohol consumption rates were reported for 28 of the 144 patients (19.4%). None of the patients admitted any consumption of controlled substances.

### Clinical findings

As mentioned in the Methodology section, patient responses were used as points of reference for different comparisons. Somatic symptom SSI patient self-reports were correlated to psychiatrists' somatic symptom reports, which the physician recorded during the patient interview by using the SSI as a general reference. All the patients included in the analysis reported at least one somatic symptom in the SSI. A low agreement between the psychiatrist report and his patients for somatic painless symptoms was observed in both visits. The Kappa values obtained for each painless somatic symptom were less than 0.1159 (low degree of agreement) with variable sensitivity ranged from 1.27% for walk balance difficulty to 18.81% for muscle twitches. In other words, the probability of psychiatrists reporting the presence of any somatic painless symptom in their patients with a MDE, which reported having this symptom, was less than 19% in this sample. In other way, a variable percentage of false negative were also reported, ranged from 81% for muscle twitches to 100% for lump in throat, meaning that near all painless somatic symptoms were erroneously interpreted by the psychiatrist as present in some cases, but they were not reported by the patient (Table 2). The total average number of painless symptoms reported by the psychiatrists was only an eighth of the number reported by the patients (2 vs. 16; p < 0.001). The number of painless somatic symptoms reported by the patient from baseline to endpoint visit was similar, showing consistency and no improvement despite treatment of their MDE (p = 0.999). No significant differences in the number of painless somatic symptoms were observed when data was adjusted for gender or age.

**Table 2:** Relationship between the treating psychiatrists and the patients reports of painless somatic symptoms

**SSI Symptom**	Kappa Coefficient*	**N**	**Sensitivity (%)†**	**False negative (%)†**
Nausea/Vomiting	0.1159	131	12.70	87
Constipation	0.0594	128	6.85	93
Dizziness	0.0521	133	10.75	89
Muscle twitches	0.0512	133	18.81	81
Breathlessness	0.0418	133	6.58	93
Fatigue	0.0261	133	9.48	90
Weakness	0.0225	133	4.85	95
Palpitations	0.0201	131	6.33	93
Lump in throat	0.0151	133	NA	100
Walk balance difficulty	0.0105	135	1.27	98
Head/nose fullness	0.0085	133	1.92	98
Body Numbness	0.0042	134	3.19	96
Other Symptoms	0.0000	132	NA	NA

*Kappa Coefficient: <0.75 = high degree of agreement beyond chance; 0.40 to 0.75 = fair agreement beyond chance; <0.40 = low degree of agreement. †For major explanation regarding sensitivity and false negative items please refer to the principal text.

NA = Non applicable.

With regards to the painful somatic symptoms, contrary to the painless ones, a fair agreement between the psychiatrist report and his patients was observed for upper limbs joint pains, abdominal pain, back pain and headache (0.4008 < κ >0.5788). Variable sensitivity ranged from 46.7% for shoulder pain to 77.2% for headache, this being one of the symptoms with higher sensitivity. Other painful symptoms, including pain in the lower limbs and joints, showed a low degree of agreement with lower sensitivity values. With regards to the percentage of false negatives, these ranged from 22% for headache to 66% for lower limb pain (Table 3). The number of painful symptoms reported by psychiatrists was half that reported by their patients (1.5 vs. 3; p < 0.001). The number of painful symptoms reported by patients was similar in both visits showing little change despite treatment of their MDE (p = 0.1453). No significant differences in the number of painful symptoms were observed when data was adjusted for gender or age.

**Table 3:** Relationship between the treating psychiatrists and the patients reports of painful somatic symptoms

**Type of Pain**	Kappa Coefficient*	**N**	**Sensitivity (%)†**	**False negative (%) †**
Hand Pain	0.5788	125	64.52	55
Abdominal Pain	0.5710	125	57.14	42
Shoulder Pain	0.5303	125	46.67	53
Back Pain	0.4180	125	62.71	37
Headache	0.4008	125	77.22	22
Knee pain	0.4034	125	39.13	60
Lower limb pain	0.3826	125	33.33	66
Foot Pain	0.3736	125	44.44	55
Other Pains	0.00 – 0.32	125	6.8 – 28.5	71 – 95

*Kappa Coefficient: <0.75 = high degree of agreement beyond chance; 0.40 to 0.75 = fair agreement beyond chance; <0.40 = low degree of agreement. †For major explanation regarding sensitivity and false negative items please refer to the principal text.

In spite of minimal changes in the number of somatic symptoms between both study visits, we observed some changes in the patients' degree of discomfort according to the SSI scores. Some symptoms exhibited a little but significant score reduction, although not enough to be considered absent after the 8 weeks of treatment (Table 4).

The data obtained from the SSI in the initial visit allowed us to observe certain characteristics common to our population sample. For example, one hundred percent of the patients reported some type of somatic symptom associated with their major depressive episode. This percentage did not vary after an average of 8 weeks of treatment with different antidepressants selected by the treating clinicians. Age, female gender, and unemployment were variables significantly associated with the presence of somatic symptoms (p < 0.05). The severity of the depression according to the patient self-evaluation was greater in those patients with moderate to high degree of discomfort with their somatic symptoms (p < 0.001) (Table 5).

**Table 5:** Sample characteristics at initial visit according to the total SSI score*

**Variable**	**Minimum degree of discomfort (n = 34)¶**	**Moderate/High degree of discomfort (n = 87)¶**	**p-Value**
**Average SSI Score **(st.dev.)	41.0 (7.7)	76.06 (16.65)	<0.001

**Average Age **(st.dev.)	40.69 (13.23)	45.5 (10.00)	0.0193

**Gender:**			
Female (77.9%)	29.25	70.75	0.034

**Employment Status:**			
Employed (58.5%) §	41.30	58.70	0.013

**Antidepressants:**			
SSRIs (61%)† T	34.88	65.12	0.553
Other antidepressants	29.03	70.97	
(26.5%) †			

**Total VAS Score ‡**	49.8	70.8	<0.001

*SSI – Somatic Symptom Inventory; §Includes housewives and students; T Includes fluoxetine, setraline, paroxetine, citalopram; † Includes venlafaxine and bupropion; ‡ Self-evaluation analogue visual scale of the depression scored by the patients; ¶Minimum degree of discomfort due to somatic symptoms (SSI ≤ 52 – 26 items); Moderate/high degree of discomfort due to somatic symptoms (SSI > 52 – 26 items).

With respect to emotional symptoms, in the first visit only 17.5% of the patients were classified with a mild clinical depression by the psychiatrists, and the average score of the total CGI-S was 4.3. This suggests a moderate to high level of severity of depression for the study sample in general. Likewise, all individual emotional symptoms were reported as moderate to high severity. Slightly more than 35% of the patients reported moderate to severe feelings of guilt and/or hopelessness according to his psychiatrist. Thirty-eight percent reported moderate to severe anhedonia, 34.3% psychomotor changes, 42.4% loss of concentration, 31.9% significant levels of anxiety, 36.1% psychotic symptoms, and 31.9% suicidal thoughts.

At the end of 8 weeks of treatment with various antidepressants, there was a significant reduction in the basal score of the total CGI-S and in each of the depressive symptoms analyzed (p < 0.0001). Nevertheless, the average score in visit 2 was 3.2 and 38.1% of patients maintained moderate to high levels of severity. Only 13% of the patients achieved a score of ≤ 2 on the CGI-S (borderline depression or normal). Patients also reported feeling better with antidepressant treatment during visit 2 as reflected by a reduction in the total average PGI-I score (3.22 to 2.73; p < 0.0019) and the VAS score (from 0.63 to 0.49 p < 0.0001). No significant statistical differences in visit 2 VAS scores were noted between patients who were already on antidepressant therapy in visit 1 (87.5%) and those who were not, but this finding could be due to the sample size.

In general, although depressive symptoms decreased significantly in visit 2 compared to visit 1, a similar trend was not detected for somatic symptoms. No improvement was noted in the painless somatic symptoms as reflected by visits 1 and 2 SSI subscale scores (51.6 and 52.5 respectively; p = NS). Slight reduction in the degree of discomfort due to the following somatic symptoms was noted: dizziness, fatigue, muscle twitches, poor physical health, body numbness, weakness and "feeling bad". However, there were no appreciable changes in gastrointestinal, sensory and cardiopulmonary symptoms during the 8 weeks of treatment. Finally, slight but significant changes were noted in visits 1 and 2 SSI subscale scores for painful symptoms (13 and 11, respectively; p < 0.05) and total SSI scores (63.4 and 56.8 respectively; p < 0.05).

**Figure 1:**
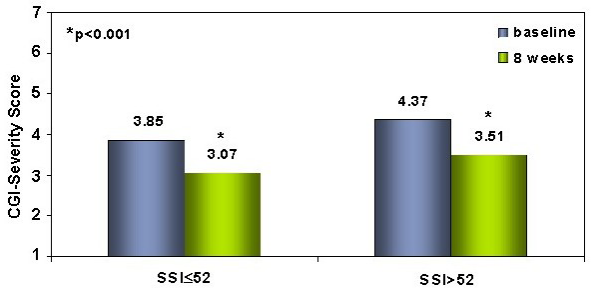
**Analysis of the SSI total score related to the mean CGI-S score**. The patients were divided into two groups according to their degree of discomfort with their somatic symptoms: minimum (≤ 52) (n = 29) and moderate/high (>52) (n = 50). CGI-S is measured from 1 to 7, where "1" corresponds to the absence of depressive symptoms and "7" to the greatest possible severity of depressive symptoms. Abbreviations: SSI – Somatic Symptom Inventory; CGI-S – Clinical Global Impression of Severity. *p < .001

**Figure 2:**
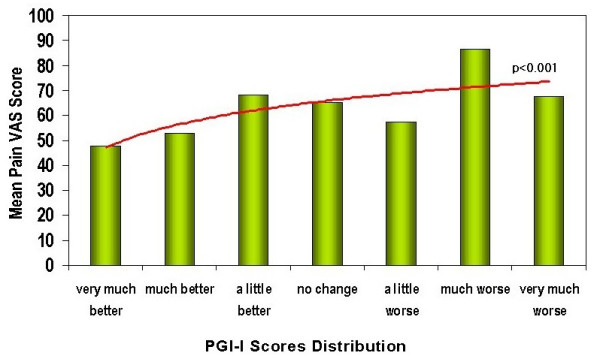
**Relationship between pain severity and depression improvement (patient report)**. Regression analysis shows an inversely proportionate relationship between the severity of the pain reported by the patients and the level of improvement from the major depressive episode according to the patients' global impression (PGI-I) (n = 87). Abbreviations: VAS – Visual Analogue Scale; PGI-I – Patient Global Impression of Improvement.

The relation between the patients' degree of discomfort with their somatic symptoms and the severity of their emotional symptoms was also analyzed. This study did not reveal any relation between the patients' level of response to antidepressant treatment and their degree of discomfort due to somatic symptoms (painless or painful) in visit 1. Patients with minimal as well as those with moderate to high degree of discomfort due to somatic symptoms demonstrated significant reductions on the CGI-S at the end of 8 weeks of treatment (Figure 1). Slight but significant reductions in depression and painful symptoms severity according with the Pain relief Visual Analogue Scale and the PGI-I – both of which were rated by the patients – were observed after 8 weeks of treatment. A proportional relationship between the pain and depression severity was observed (p < 0.0001) (Figure 2).

## Discussion

Somatization is common to all cultures and social groups studied. However, there are differences in the styles of expression and attribution of symptoms according to the beliefs and health practices of each culture [35-38]. Somatization has been more frequently associated with Latino populations than with other ethnic groups [31]. It has been mentioned that societies that promote individualism with clear limits in interpersonal relations seem to value the direct expression of unpleasant feelings. On the other hand, collective societies with more flexible levels of relationships tend to place greater value on the indirect expression of feelings (idiom of distress hypothesis) [39]. The Psychological Problems in the General Health Care study (PPGHC) of the World Health Organization (WHO) was conducted in 14 cities from the same number of countries on four continents. It showed that adult patients treated in primary outpatient care centers were characterized by a high prevalence of reports of somatic symptoms in the two participating Latino cities (Rio de Janeiro in Brazil and Santiago in Chile), compared to the total sample (32% and 36.5% vs. 19.7%), according to the SSI scale. These results did not depend on the level of development, education, or gender of the cities evaluated [40]. The WHO study did not demonstrate culture to have a determining role in the manifestation of somatic symptoms except for the two Latin American cities evaluated.

Cultural differences can even be observed within different Latino populations in the prevalence of somatic symptoms or chronic pain. A comparative study of Mexican-Americans, natives living in Puerto Rico, and Caucasian-Americans used an analysis of 5 clusters of symptoms and demonstrated that the Puerto Ricans presented the highest levels of somatization [25,41]. In other publications, the same authors concluded that since the high rates of somatization in Puerto Ricans were not accompanied by higher rates of prevalence of depressive disorders compared with the US, Puerto Rican physicians were likely attributing somatic symptoms as "psychogenic" and not considering them manifestations of a MDE [42,43]. Another study of depressed patients living in the United States reflected prevalence rates of chronic abdominal pain that differed significantly between the three Latino groups evaluated: 4.6% in Mexican-Americans; 5.8% in Cuban-Americans; and 8.3% in Puerto Ricans. Logistic regression analyses showed a close relation between depression and chronic abdominal pain, female gender, and single marital status [44].

In our study, a direct relationship between moderate to high degree of discomfort due to somatic symptoms and age, female gender and unemployment was also observed (Table 5). In this study of Puerto Rican patients with a MDE, 100% of patients reported somatic symptoms using the SSI scale. Although this percentage is very high, previous depression trials in primary care settings have also shown that 69% to 92% of MDE patients experience somatic symptoms [8-11]. Somatization has been reported to be particularly more prevalent in Puerto Rican depressed patients than in other Hispanic populations. However, we cannot exclude the possibility that the use of an inventory such as SSI led to a higher number of somatic symptom reports than would have been spontaneously reported by patients. We could have avoided this potential bias by using a validated scale to collect information on somatic symptoms as a primary research tool. However, this would have precluded us from achieving the primary objective of our study.

Communication between patients and their physicians is influenced by various cultural factors such as the patient's perception of her emotional and physical symptoms, her report of these symptoms to the physician, and the physician's interpretation of the symptoms. These steps described by Leff [45] in the communication of unpleasant emotions could affect the final evaluation of the physician with respect to the clinical status of the patient. Since 1960, significant differences have been shown between patient self-evaluations of their depressive symptomatology and the evaluations conducted by their physicians. Carrol et al. [46] suggest that these differences are due to discrepancies between the instruments used by the patients and clinicians. They proved that the use of instruments with similar structure and matching items allow higher correlation coefficients. Corruble et al. [47], using structured instruments with similar items, demonstrated in their study with 64 hospitalized depressed patients that there was significant agreement between the patients and the psychiatrists' report of the severity of the depression. Nevertheless, depressed patients with high levels of somatization and anxiety exhibited a tendency to overestimate their symptomatology compared to what was reported by their physicians. To date, few studies have explored the level of agreement in the recognition of somatic symptoms between patients and physicians. A study with primary care physicians showed that these clinicians and their patients can be more comfortable with somatic symptoms than with emotional symptoms, which leads to an under-reporting of depression in those patients with associated physical symptoms [48]. Contrary to this, a review focused on the recognition of somatoform disorders by psychiatrists indicated that these specialists are more concerned with severe mental disorders than with the recognition of somatic symptoms. Psychiatrists attempt to "normalize" the physical symptoms expressed by their patients and tend to refer them to other specialists [49].

In this study, despite similar SSI mean scores for painful and painless symptoms (2.6 vs. 2.5, respectively) (Table 4), when compared to painful symptoms of the torso, upper limbs and head, painful symptoms of the lower limbs and painless somatic symptoms showed a lack of correlation between the psychiatrists' and the patients' reports. The marked difference between psychiatrists' and patients' somatic symptoms reports may be explained by the different methods used to document somatic symptoms and degree of discomfort caused by them, or may be due to a tendency by psychiatrists to dismiss certain type of pain or painless somatic symptoms in their usual clinical practice. This study was purposefully designed to avoid any intervention and allow for a naturalistic observation of usual clinical psychiatric care in Puerto Rico. The use of a structured symptom checklist for psychiatrists would not have been representative of the usual clinical practice and would not have allowed us to document the degree of recognition of somatic symptoms in MDE patients in this clinical setting.

Additionally, we observed that those somatic symptoms with lower sensitivity percentages usually have higher false negative percentages than those with fair agreement levels (Tables 2 &3). This suggest that psychiatrists may not only omit several somatic symptoms in their patients with MDE but may also detect and document other symptoms unreported by their patients. This observation may be a result of the study design, which in itself could have increased the psychiatrists' motivation to report somatic symptoms and may not represent necessarily their usual clinical practice.

Failure to detect somatic symptoms in depressed patients may have significant implications in the cost of treatment. Reid et al. [50] compared the health-care utilization patterns of patients with medically unexplained symptoms with those of other frequently referred patients. "Somatising patients" had at least two medical consultation visits for unexplained physical symptoms, a greater number of referrals for secondary care and were more likely to undergo additional clinical work-up and tests. In other trials, rates of health resources utilization for MDD patients with somatic symptoms were nine times higher than for the general population and three times higher than for all depressed patients [51,52]. Moreover, individuals with MDD consistently exhibit the highest rates of loss of productive time (LPT) when they concomitantly present symptoms like pain, weakness, fatigue, gastrointestinal discomfort or sensory changes [53]. Given the duration and size of the sample, this study did not show differences in certain variables related to the use of health resources such as consumption of analgesics or other medications. However, it did reflect a higher rate of unemployment in patients with moderate to high severe somatic symptoms.

Additionally, failure to detect somatic symptoms in depressed patients has significant impact in the response to treatment. The presence of physical symptoms in MDD patients has been associated with a poor level of response to antidepressant treatment [54]. Appropriate treatment to control non-emotional symptoms is essential in order to achieve proper compliance with therapy [55], to decrease the risk of recurrences [56], to maximize earlier initiation of the antidepressant action [57], and to increase the opportunities for complete remission of the depressive episode [58]. In this study, although there was a significant reduction in the average CGI-S depression score, only 13% of the patients reached a level of remission defined *post-hoc *as CGI ≤ 2.

Several studies with Selective Serotonin Reuptake Inhibitors (SSRIs) have demonstrated that depressed patients with somatic symptoms exhibit lower rates of response than those without somatic symptoms [54,59,60]. Additionally, somatic symptoms respond less to SSRIs than non-somatic symptoms [61]. According to several authors [7,62,63], antidepressants that act dually on the noradrenaline and serotonin pathways could be better candidates for treating both depression and concomitant somatic symptoms. In this study, 61% of the patients were treated with a SSRI and 13% with low doses of *venlafaxine *(average 56 mg/day). Clinicians did not report differences, according to the CGI-S, in the level of antidepressant response between patients with minimal and those with moderate to high degree of discomfort with their somatic symptoms. Nevertheless, it was possible to determine that those patients who reported less relief of painful symptoms exhibited lower levels of reduction in the PGI-I for depression. In other words, although psychiatrists were not able to observe significant differences in the level of response to treatment, patients who had less relief of painful symptoms reported a lower response to antidepressant treatment (Figure 2). This difference in the reporting of the response to treatment could be due to the emphasis placed by psychiatrists on detecting and controlling emotional symptoms rather than somatic symptoms. It also suggests that inappropriate identification of somatic symptoms may lead to an erroneous perception of appropriate antidepressant treatment response vis-à-vis the experience of the patient.

Our study has a number of strengths. Through a confirmatory analysis, we explicitly tested the level of recognition of somatic symptoms in patients with a MDE by their psychiatrists. This analysis was conducted for all the somatic symptoms as a group and separately according the presence of pain or not. We studied a random sample of the population, and we used valid and reliable measures.

One potential limitation in our study is the use of the SSI, which is a valid tool to detect the presence and degree of discomfort due to somatic symptoms. However, the SSI is probably more predictive in the case of anxiety disorders than in depression and has a reduced sensitivity in the detection of symptom changes due to treatment [64]. Another possible limitation is our use of a spontaneous report measure for somatic symptoms by psychiatrists. A structured psychiatric interview may have yielded different results. However, spontaneous reports were the only way not to bias the researchers' responses. In addition, patient reports constituted the gold standard for the analysis of data despite the absence of scales or figures to guide the patient in his response, potentially yielding an incomplete report of symptoms.

The type of sampling of patients used could also be a limiting factor. Although the study attempted to reproduce as faithfully as possible the usual psychiatry practice in Puerto Rico with appropriate representation throughout the Island, exclusion and inclusion criteria limited access to hospitalized patients with refractory depression, suicidal behavior and associated medical conditions. These are well-known factors that increase the presence of physical symptoms in patients with major depression [14,17]. Only Latino patients living in Puerto Rico were evaluated. Given the importance of cultural factors in measuring the objectives set forth in this study, it will be difficult to generalize the data to other Latino populations and even less so to other ethnic groups or cultures.

Another potential limitation is the size of the sample. On the one hand, it made possible the detection of significant differences between psychiatrists and their patients with a MDE in the reporting of physical symptoms, but it did not allow confirmation of the impact of antidepressants in the control of physical symptoms. A dichotomous classification of the clinical response of emotional and physical symptoms rather than a continuous measurement of their evolution was used because of the limited sample size.

Finally, the short period of time between the two visits (8 weeks), the absence of intermediate visits and the lack of treatment compliance measurements may limit conclusions on the response to antidepressant therapy.

## Conclusion

In summary, these results confirm reports from previous clinical trials about the high rate of somatic symptoms in Puerto Rican patients with a MDE [25,41,44]. Collectively, our data reflect a significant difference in the report of several somatic symptoms – pain in lower limbs and joints and painless symptoms – by the psychiatrists and their patients. Age, female gender, and unemployment are variables significantly associated with the presence of somatic symptoms. The severity of the depression according to the patient self-evaluation is greater in patients with moderate to severe discomfort due to somatic symptoms.

Although depressive symptoms in general reflected a significant reduction in visit 2 compared to visit 1, 38.1% of patients still had moderate to high severity depression and only 13% had CGI-S scores of ≤ 2 (borderline or normal depression). Slight but significant reductions in depression and painful symptoms severity were observed according to the patient evaluations after 8 weeks of treatment. However, a proportional inverse relationship between the pain relief and depression severity was observed. This finding suggests that physical symptoms must be appropriately recognized by psychiatrists because they can interfere with the physicians' assessment of the magnitude of response to antidepressant treatment.

## Competing interests

This work was sponsored by Eli Lilly and Company, San Juan, Puerto Rico.

Dr. Tamayo & Rivas are full-time employees and hold shares of Eli Lilly & Co. (San Juan, Puerto Rico). Mrs. Roman was a statistician contractor of Eli Lilly & Co. (San Juan, Puerto Rico). Dr. Fumero has received research support and/or honoraria from Abbott, Astra Zeneca, Bristol-Myers Squibb, Eli Lilly, Glaxo Smith Klaine, Janssen, and Wyeth-Ayers.

## Authors' contributions

JT conceived and designed the study and the study clinical report forms, was involved in the drafting the article and the interpretation of the data. KR participated in the study design, made the statistical analyses, helped to draft the manuscript, and was involved in the interpretation of the data. JF participated in the acquisition of the data and revised the article for intellectual content. MR made substantial contributions to the conception of the article and revised the article for intellectual content. All authors read and approved the final manuscript.

## Pre-publication history

The pre-publication history for this paper can be accessed here:


